# Research on Thermal Runaway Monitoring Methods for Lithium-Ion Batteries Based on Continuous Acoustic Emission Technology

**DOI:** 10.3390/s26134130

**Published:** 2026-06-30

**Authors:** Bingxi Liu, Fumin Li, Xiaoyang Bi, Xiao Ma, Cuihua An, Qibo Deng, Ping Zhuo

**Affiliations:** 1 The Key Laboratory of Fire Protection Technology for Industry and Public Building, Ministry of Emergency Management, Tianjin 300381, China; liubingxi@tfri.com.cn (B.L.);; 2 Tianjin Fire Science and Technology Research Institute of MEM, Tianjin 300381, China; 3 School of Mechanical Engineering, Hebei University of Technology, Tianjin 300401, China; lifumin88920@163.com (F.L.); maxiao1232025@163.com (X.M.); ancuihua@hebut.edu.cn (C.A.);

**Keywords:** continuous acoustic emission, convolutional neural network, lithium-ion battery, thermal runaway warning

## Abstract

Lithium-ion batteries (LIBs) are widely used; however, they have safety hazards because of their susceptibility to thermal runaway (TR). Current early warning methods rely on the external monitoring of parameters such as temperature and strain. These methods have an inherent lag, as the signals can only be detected after internal heat and gas accumulation. Internal sensors are difficult to implement due to the harsh environment and high cost, leaving the ultra-early incubation stage of TR poorly addressed. To overcome these limitations, this study introduces acoustic emission (AE) technology for the real-time external detection of internal TR reactions. An experimental platform induced TR through overcharging, integrating multi-source AE and temperature signal acquisition. Continuous AE signals were collected from the onset of overcharging until the valve opened. Time–frequency analysis revealed anomalous waveform features in the early stage of TR; a two-dimensional method enhanced frequency-domain recognition. Combining the processed AE signals with a convolutional neural network achieved high-accuracy phase segmentation. Cross-validation and comparisons with temperature-based methods demonstrate the effectiveness and precision of AE monitoring for ultra-early TR warning. The results highlight the potential of AE-based monitoring as a proactive risk-management strategy, supporting dynamic assessment and safety responses in energy-storage applications.

## 1. Introduction

As energy crises and environmental pollution issues intensify, developing clean energy has become a global consensus [1]. Lithium-ion batteries (LIBs) are widely used in new energy vehicles, electric vehicles, and energy storage stations due to their high energy density, strong performance, long service life, and compact size [2,3]. However, incidents involving thermal runaway (TR) caused by improper use of LIBs continue to pose threats to life and property safety [4].

TR in LIBs refers to a chain-reaction phenomenon triggered by internal short circuits under conditions of electrical, thermal, or mechanical abuse [5,6]. This leads to continuously rising battery temperatures, the release of toxic gases, and even combustion or explosion [7]. To mitigate and prevent the dangers posed by lithium-ion battery TR, numerous researchers have analyzed the internal mechanisms of TR in LIBs and developed corresponding early warning strategies. TR in LIBs is a complex electrochemical process [8], primarily involving the decomposition and regeneration of the solid electrolyte interphase (SEI) film, the decomposition and evaporation of cathode/anode materials, separators, and electrolytes, as well as reactions between cathode/anode materials and electrolytes [9]. As these reactions progress, the battery exhibits abnormal changes in temperature, voltage, internal resistance, expansion force, and gas generation. Analyzing the patterns of these parameter changes during TR enables the identification of early critical points, allowing for the classification of corresponding stages and the issuance of warnings. This represents the mainstream approach to lithium-ion battery TR early warning systems [10].

Acoustic emission (AE) technology has recently emerged as a promising non-invasive technique for monitoring internal reactions in LIBs throughout various abuse scenarios. Prior studies have demonstrated that AE signals can effectively detect battery defects, overcharge-induced degradation, and casing swelling in real time [11,12,13]. For instance, continuous energy release during mechanically induced TR in LIBs has been characterized using AE monitoring, revealing distinct AE signatures corresponding to internal fracture and gas-evolution events [11]. Similarly, AE-based approaches have been applied to sodium-ion batteries to track performance degradation under overcharge conditions [12], and to lithium-ion cells to simultaneously identify defects, overcharge onset, and mechanical expansion behavior [13]. However, these studies predominantly rely on parametric AE analysis, which detects only discrete impact events exceeding a preset threshold, thereby missing the continuous low-level AE activity that characterizes the early incubation stage of TR. Furthermore, a systematic classification of the full TR progression into multiple stages using continuous AE signals, combined with deep learning, has not been reported. The present study addresses these gaps by analyzing full continuous AE waveforms from overcharge onset to valve opening and integrating a two-dimensional frequency-domain feature extraction method with a convolutional neural network (CNN) to achieve high-accuracy, six-stage TR phase segmentation.

Methods for TR early warning can be categorized as external and internal, based on sensor placement. External monitoring deploys sensors on the battery casing to collect signals reflecting macroscopic phenomena such as expansion, temperature rise, and gas generation. Among these, temperature is the most conspicuous parameter and currently serves as the mainstream monitoring approach. Song et al. [14] identified a critical surface temperature of 125 ± 3 °C for TR occurrence in LFP batteries. Additionally, side reactions during the early stages generate gases that cause battery expansion. Li et al. [15] and Lin et al. [16] found that expansion force signals exhibit abnormal characteristics earlier than voltage and temperature signals. Zhang et al. [17] and Yang et al. [18] demonstrated that hydrogen appearance precedes TR by hundreds of seconds. However, external monitoring methods capture only the cumulative results of internal reactions, such as heat propagation and gas accumulation, which require a certain delay before causing significant changes in surface sensor signals. However, external parameters, such as terminal voltage and surface temperature, merely reflect the aggregated outcomes of complex internal electrochemical–thermal interactions, introducing inherent response delays that limit their effectiveness for early warning [19]. For instance, studies have shown that internal temperatures can exceed external readings by hundreds of degrees during thermal runaway, rendering surface temperature readings inadequate for timely safety assessment. Similarly, gases require sufficient pressure to accumulate and breach the safety valve. By the time valve opening occurs—a detectable event for existing sensors—the battery is already in a dangerous state, with internal exothermic reactions becoming difficult to contain, potentially leading to combustion or explosion within a short timeframe [20].

Internal monitoring embeds sensors within the battery during manufacturing to collect and transmit data, enabling effective detection of internal abnormal reactions during TR. Li et al. [21] embedded fiber Bragg grating (FBG) optical sensors within lithium-ion batteries to monitor internal pressure and temperature changes, establishing thresholds for TR risk assessment. Jia et al. [22] placed internal thermocouples in LIBs under varying ambient temperatures and charging rates. Based on internal temperatures, they proposed that exceeding 62 °C—the threshold for SEI film decomposition—constitutes the first stage of TR warning. While internal monitoring can, theoretically, provide earlier warning than external methods, it faces substantial challenges from a process safety engineering perspective. The implantation of sensors requires perforation of the battery’s monolithic encapsulation, which can significantly impact normal service life and introduce potential leak paths [23]. Signal transmission demands advanced packaging techniques and stringent signal shielding, further elevating process, cost, and reliability demands. Additionally, the potential for interference between internal sensor electrical signals and internal battery reactions remains uncertain, posing significant challenges for practical engineering implementation. A comparison of the various methods is shown in Table 1.

This study introduces a detection technology and proposes a TR early warning method that enables real-time monitoring of internal abnormal reaction data signals from outside the battery. Acoustic signal detection technology combines the advantages of both external and internal monitoring. Although sensors are positioned outside the battery, they can still provide real-time and efficient feedback on the battery’s internal condition. Acoustoelastic wave signals generated by internal damage and abnormal events within the battery are transmitted to the AE sensor on the battery surface. Compared to the propagation time of acoustoelastic waves during the TR process, this transmission time is negligible. By analyzing elastic wave signals collected from the battery surface that reflect internal reactions, this study explores the time and frequency-domain characteristics of AE signals during the early stages of TR induced by overcharging in LIBs. This enables precise classification of different TR stages before valve opening, offering a novel approach to identifying and warning against the early incubation phase of TR caused by overcharging in LIBs.

The main contributions of this work are threefold: (1) continuous AE signals rather than discrete parametric events are systematically analyzed across all six TR incubation stages before valve opening, providing a more comprehensive characterization of the full TR evolution; (2) a novel two-dimensional frequency-domain feature extraction method is proposed, which converts one-dimensional continuous AE spectra into grayscale image samples, enabling effective CNN-based multi-stage classification; and (3) the proposed method is quantitatively shown to provide early warning approximately 374 s earlier than surface temperature-based methods, demonstrating its superiority for ultra-early TR incubation detection.

## 2. Materials and Methods

AE detection is a passive technique that captures elastic waves generated by internal reactions within materials or structures [24]. During normal battery operation, lithium-ion motion generates low-energy elastic waves, whereas TR triggers stronger signals from side reactions such as SEI film decomposition, material cracking, and gas evolution. AE technology is mainly divided into parametric and continuous approaches. Parametric analysis relies on preset thresholds to identify impact events. Still, it tends to miss continuous abnormal processes, such as gradual SEI degradation, that do not exceed those thresholds, thereby losing critical frequency-domain information. In contrast, continuous analysis captures all elastic wave signals and enables full time–frequency characterization, making it better suited for monitoring the entire TR evolution. Therefore, this study focuses on continuous AE signals to investigate the progression of internal reactions throughout the TR process.

### 2.1. Experimental Methods

**Figure 1:**
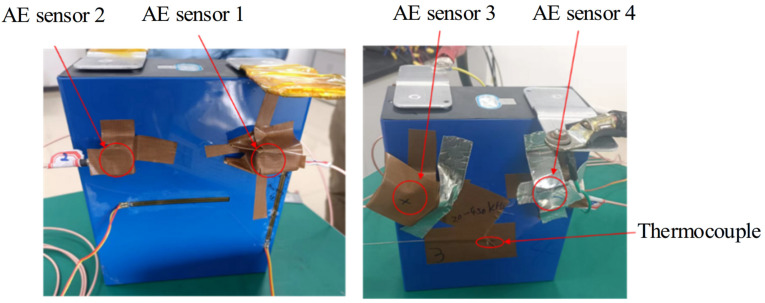
Position diagram of AE sensors on both sides of the battery.

This experiment used a 280 Ah prismatic battery for energy storage. The battery dimensions were 170 × 70 × 200 (mm), and it featured LFP cathode material and graphite anode. As shown in Figure 1, four AE sensors were positioned closer to the internal electrodes to capture acoustic signals with higher energy intensity and greater effectiveness.

**Figure 2:**
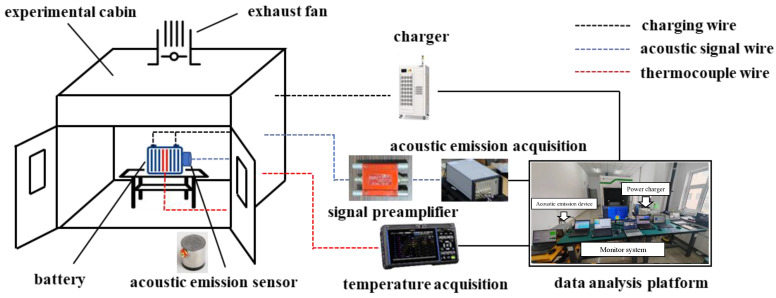
Schematic diagram of the experimental bench layout.

As shown in Figure 2, for safety, the experiment was conducted in a dedicated test chamber equipped with a large fan at the top to prevent toxic and harmful gases emitted during the experiment, and the battery was placed inside an explosion-proof container. AE signals were collected using the AMSY-6 AE system manufactured by Vallen GmbH of Germany (Munich, Germany) with the VS45-H sensor and AEP4 amplifier. The AEP4 preamplifier provided a fixed gain of 34 dB, raising the signal level well above the ADC noise floor prior to digitization. The AMSY-6’s 16-bit A/D converter at 10 MHz sampling yields a dynamic range of approximately 96 dB (theoretical), sufficient to resolve AE amplitudes spanning from ambient noise levels (~0.005 mV) to large TR-induced bursts (~2 mV). The minimum resolvable amplitude step at the ADC input is approximately 0.03 μV after preamplification, which is negligible compared to the smallest TR-characteristic signal features identified in this study (≥0.005 mV). Consequently, ADC quantization effects were not expected to compromise the frequency-domain feature extraction used in the proposed method. The sensor’s effective bandwidth ranged from 20 kHz to 450 kHz, and the system was controlled via a computer running Vallen Control Panel software R2022.0809.3. To ensure comprehensive, high-quality signal acquisition, the sampling rate was set to 10 MHz.

The experimental procedure comprised the following steps:

1. Battery preparation: The cell was fully charged to 100% state of charge (SOC) at a 0.5C constant-current constant-voltage (CC-CV) protocol, followed by a 30-min rest period to ensure electrochemical stability;
2. Sensor mounting: Four VS45-H AE sensors were coupled to the battery surface using vacuum grease as the couplant and secured with elastic bands to ensure consistent acoustic contact. Sensor positions are shown in Figure 1;
3. System calibration: Prior to each experiment, a Hsu–Nielsen pencil-lead break test (0.5 mm, 2H) was performed at each sensor location to verify coupling quality and confirm system sensitivity within the 20–450 kHz frequency bandwidth;
4. Environmental noise characterization: AE baseline signals were recorded for 60 s before overcharge initiation. The ambient noise floor was confirmed to be below 0.005 mV in the 20–450 kHz range, well below the threshold for TR-induced AE events, ensuring that subsequent signal changes are attributable to internal battery reactions;
5. Overcharge induction: Constant-current overcharge was applied at a 1C rate from 100% SOC until safety valve opening at 556 s. No external mechanical preload was applied to the battery during testing, ensuring that the detected AE signals reflect internal electrochemical reactions rather than externally induced mechanical stress;
6. Data acquisition and export: Continuous AE waveforms and surface temperature signals were recorded synchronously. AE data were exported via Vallen Visual AE software R2022.0809.3 at a 10 MHz sampling rate for subsequent time–frequency analysis.

In order to eliminate random experimental errors and evaluate the reproducibility of observed acoustic emission (AE) characteristics, parallel repeated experiments were conducted using additional identical 280 Ah LFP battery cells. These repeated tests were conducted in two different environmental settings: (1) simulated battery module environment, and (2) simulated energy storage warehouse environment.

### 2.2. Experimental Process and AE Signal

**Figure 3:**
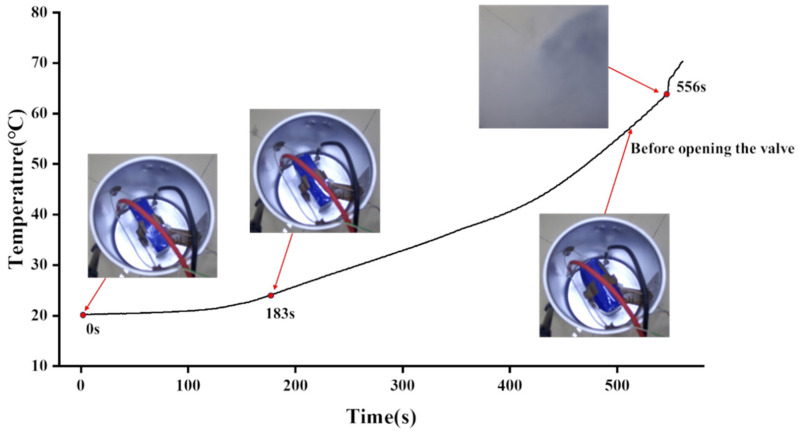
Battery shape and temperature change graph.

The battery expansion and temperature are shown in Figure 3. In the initial stage of overcharging, no significant changes occur, with temperature rising slowly at a rate not exceeding 0.08 °C/s. As overcharging progressed, the battery gradually expanded, and the rate of temperature increase exceeded 0.1 °C/s at 183 s. Subsequently, the battery continued to expand, and the safety valve opened at 556 s.

The continuous sonic emission data collected during the experiment were exported using the Vallen Visual AE analysis software, capturing 556 s of continuous sonic emission signals from battery overcharge to valve opening. This study focuses on exploring the mapping relationship between the signal characteristics during the early incubation stage of the TR process, prior to valve opening, and the TR process itself.

**Figure 4:**
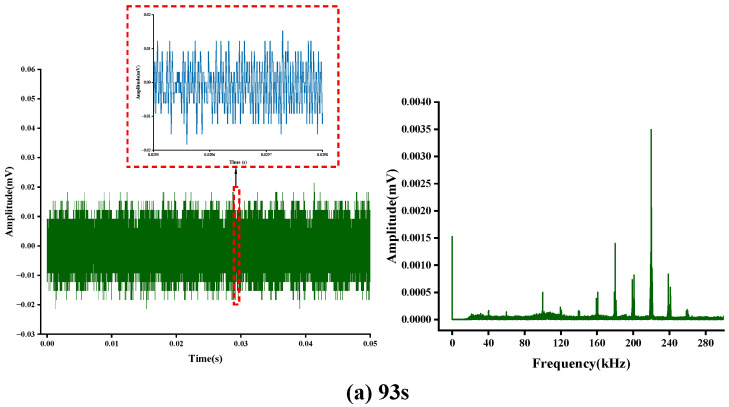

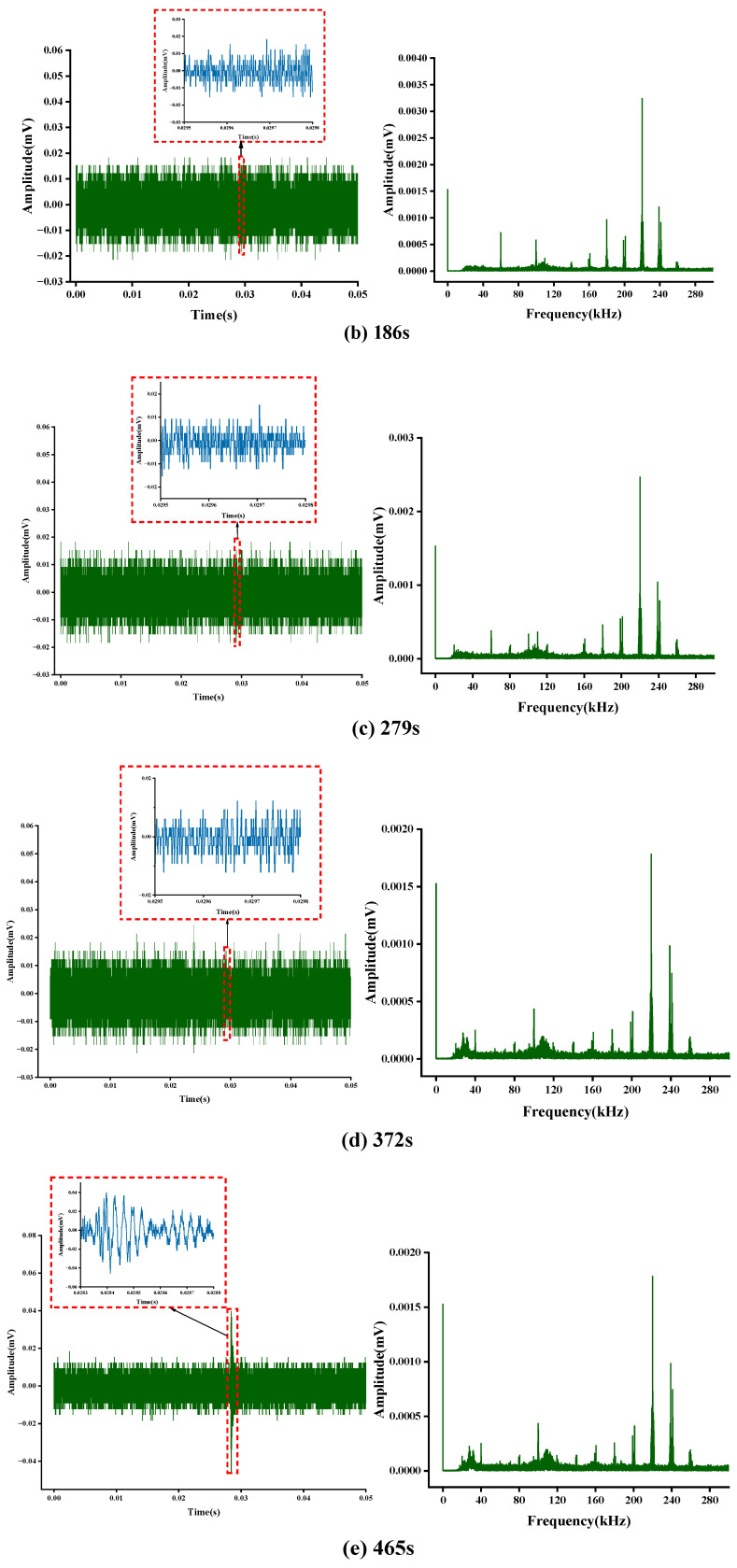

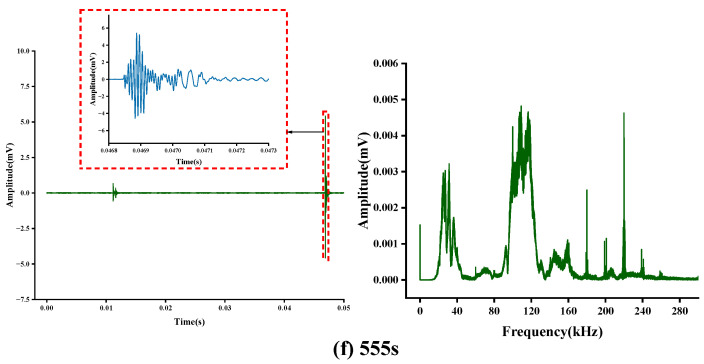
AE signal from overcharge.

After data processing and time alignment, he continued to analyze the AE signals acquired during battery overcharging to characterize their temporal evolution before valve opening. The data were subsequently divided into six equal segments based on overcharge duration, each spanning 93 s. Figure 4a–f presents the raw time-domain signals, local magnifications, and corresponding spectra of the continuous 0.05-s duration AE data collected every 93 s by Sensor 1 (Channel 1) since the onset of overcharging.

The apparent regularity of spectral amplitudes in Figure 4a–d is not attributable to ADC quantization artifacts: the preamplifier gain of 34 dB ensures that even the smallest steady-state AE signals are amplified to levels comfortably above the ADC’s least significant bit; the 16-bit resolution provides 65,536 amplitude levels across the full input range. The quantized FFT spectra therefore faithfully represent the true spectral content of the AE signals.

During the initial overcharge phase (Figure 4a–c), the AE signal exhibits stable time-domain waveforms with amplitudes consistently within ±0.02 mV. After applying a Fourier transform to a 0.05 s time-domain data segment, the frequency components remained relatively stable, revealing dominant frequencies at 220 kHz, 200 kHz, 180 kHz, 160 kHz, 140 kHz, 100 kHz, 60 kHz, and 40 kHz, with a main frequency bandwidth of approximately 4 kHz. The highest energy-associated frequency remains stable at 220 kHz, followed by 180 kHz and 240 kHz.

As overcharging progresses, defects continuously emerge within the battery structure, manifesting as abnormal impulse signals exceeding 0.02 mV in amplitude within the time-domain waveform (Figure 4d,e). During this phase, energy associated with frequency components in the ranges of 20–40 kHz and 100–120 kHz significantly increases. These impulse signals appear as abnormal waveforms in the time domain, distinct from those observed under steady-state conditions. As TR deepens within the battery, causing structural damage, the amplitude of these abnormal waveforms intensifies significantly (as shown in Figure 4f). These represent elastic waves generated by abnormal events such as the decomposition and shedding of electrode materials, the formation of gas bubbles, and the deformation of the battery casing.

## 3. Signal Analysis and Discussion

The time-domain statistical features employed in this study are grounded in signal processing theory and selected for their physical relevance to impulsive AE events associated with TR. Specifically:

Variance (σ^2^): quantifies signal power dispersion around the mean, defined as

(1) $$\sigma^{2}=\frac{\Sigma {\left(x_{i}-\bar{x}\right)}^{2}}{N}$$

An increase in variance indicates the emergence of large-amplitude AE bursts corresponding to rapid internal damage events.

Root Mean Square (RMS): reflects the average signal energy,

(2) $$RMS=\sqrt{\frac{\Sigma {x_{i}}^{2}}{N}}$$

A sustained rise in RMS indicates continuous energy release from progressive internal degradation.

Pulse Factor (*Cf*): defined as

(3) $$Cf=\frac{x_{peak}}{x_{RMS}}$$

this dimensionless metric is particularly sensitive to transient impact events. A high Cf value indicates the presence of short-duration, high-amplitude AE bursts characteristic of sudden internal fracture or gas evolution.

Kurtosis Factor (*K*): defined as

(4) $$K=\frac{\Sigma {\left(x_{i}-\bar{x}\right)}^{4}}{N\sigma^{4}}$$

this dimensionless measure quantifies the peakedness of the amplitude distribution. A Gaussian signal yields K = 3; values significantly above 3 indicate the presence of impulsive components, while values below 3 suggest a flatter distribution. In this study, K, deviating from the baseline range of 2.5–3.5, is used as a marker of abnormal AE activity.

**Figure 5:**
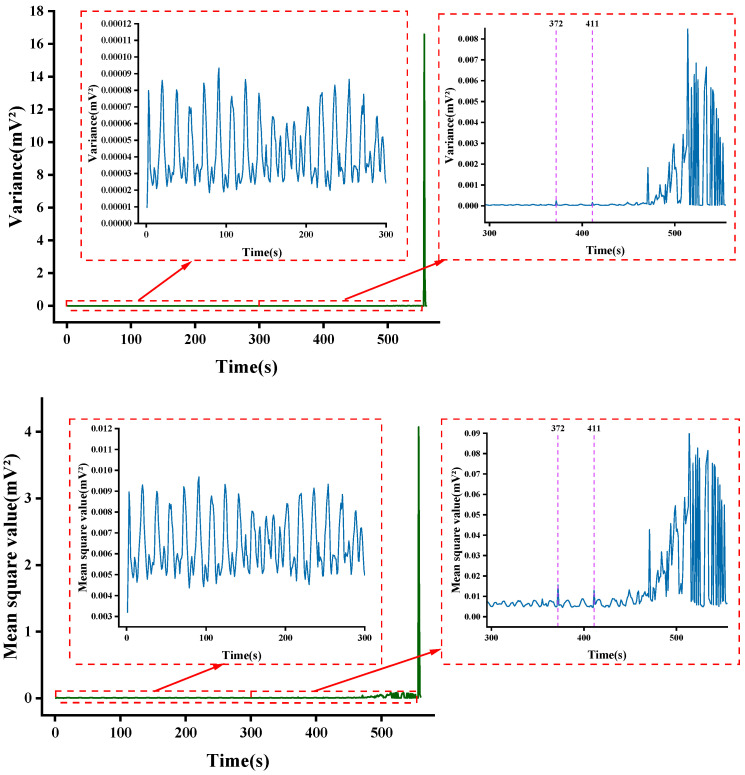
Variation in variance and mean square value of acoustic signals.

**Figure 6:**
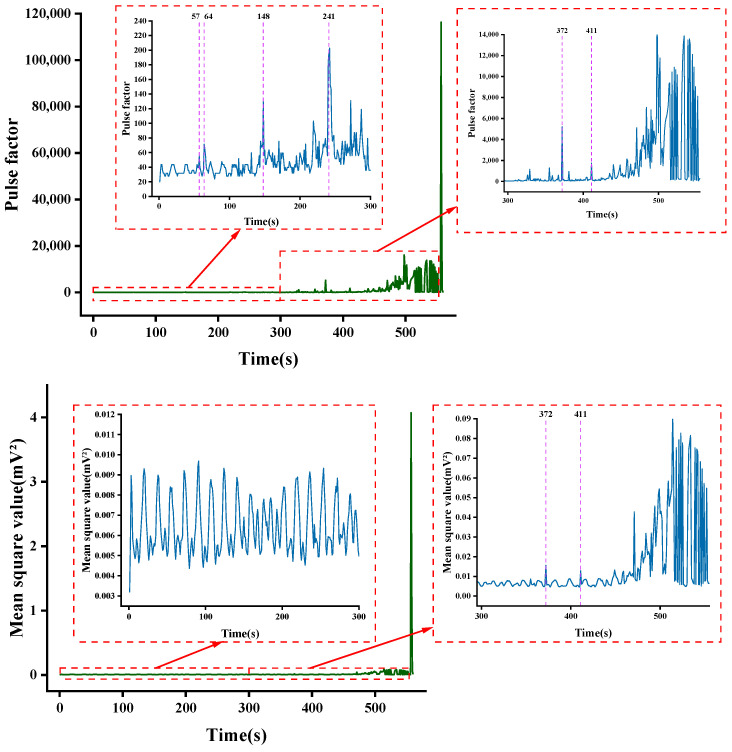
Variations in the pulse factor and mean square value of the acoustic signal.

To investigate the impact of these abnormal events on acoustic signals, a statistical analysis was conducted on the time-domain characteristics of acoustic signals recorded at one-second intervals during the battery overcharge-to-valve-opening process. Here, abnormal waveforms refer to impulse signals with amplitude variations exceeding ±0.04 mV, distinct from steady signals whose amplitude fluctuates within ±0.02 mV. Figure 5 and Figure 6 illustrate the variations in four temporal domain characteristics: variance, mean square value, pulse factor, and kurtosis factor from 0 to 556 s during battery overcharging. All four characteristics peaked at 556 s when the battery valve opened. Notably, the specific deviations observed in each feature carry distinct physical significance: elevated variance and mean square value indicate a sustained increase in overall signal energy, corresponding to progressive internal damage accumulation that generates continuous low-level acoustic activity. In contrast, sharp spikes in the pulse factor reflect the transient, high-amplitude bursts characteristic of sudden internal mechanical events such as lithium plating-induced cracking and localized separator rupture.

As shown in Figure 5, variance and RMS represent signal dispersion and average power, respectively, measured in mV^2^. Both exhibit periodicity before 439 s, with abnormal values at 372 s and 411 s. The sudden increase in these features indicates the emergence of numerous waveforms with large-amplitude variations, widening the gap between the overall signal and its average. This indicates that internal battery components or the casing began sustaining damage and releasing elastic waves.

Subsequently, between 439 s and 555 s, as overcharging progressed, the battery continued to sustain damage and release elastic waves. Abnormal values for both characteristics were observed in large numbers and were significantly higher than those observed during the initial overcharging phase. Three types of anomalous waveforms continuously appeared in the time-domain signal. At this stage, various TR-side reactions had initiated within the battery, causing a continuous temperature rise and gradual gas accumulation inside, which indicated that, as overcharging progressed, AE signals from abnormal events, such as internal cracking or gas bubbling, became increasingly distinguishable. Finally, at 556 s, the battery valve opened, releasing gas and electrolyte. The acoustic signal energy intensity reached its peak, appearing as an extremely high point in the feature evolution profile.

The other two dimensionless time-domain features, the pulse factor and kurtosis factor, exhibited greater sensitivity to signal anomalies, as shown in Figure 6. Since TR processes show significant correlation with the frequency and amplitude characteristics of impact signals, these two features were selected for analysis. The pulse factor detects impulsive components in the signal, while impact signals significantly influence the kurtosis factor. To assess signal curve kurtosis, a kurtosis value of 3 indicates normal kurtosis. Kurtosis values greater than 3 and less than 3, respectively, indicate signal peak heights above and below the normal distribution curve.

Both kurtosis and skewness exhibit abnormal values earlier than variance and mean. The pulse factor first showed high-amplitude abnormal values exceeding stable levels at 57 s and 64 s after overcharging commenced. Localized amplification of the time-domain signals at these two points revealed one second-type and one third-type anomalous waveform, respectively.

Furthermore, when the pulse factor exceeded 43, anomalous waveforms appeared during that period. Regardless of waveform type, the increase in the number of anomalous events corresponded to a higher pulse factor value. During the effective AE signal acquisition period from 0 to 556 s of overcharging, the pulse factor exceeded 43 for 394 s, accounting for 70.86% of the total time. This indicates that, beyond the relatively stable initial phase, various abnormalities had already begun developing within the battery throughout the remainder of the overcharging period. This study showed that abnormal waveforms were detected as early as 57 s into overcharging, demonstrating the sensitivity of AE monitoring in tracking battery TR progression. The steepness characteristic remained within a relatively stable range of 2.5–3.5 before 240 s, with no abnormal values. The first abnormal value appeared at 148 s, synchronized with the waveform factor. Starting at 241 s, the steepness factor and pulse factor exhibited consistent changes, validating the consistency of shock waveform characteristics. Subsequently, the amplitude of the abnormal waveform increased. As overcharging progresses, multiple TR side reactions occur within the battery. The released heat further intensifies these reactions. Crack formation, decomposition of active materials, and gas evolution, coupled with varying degrees of deformation at different locations of the battery casing, continuously generate multiple elastic waves. These waves amplify and superimpose, causing significant and violent fluctuations in the time-domain characteristics.

**Figure 7:**
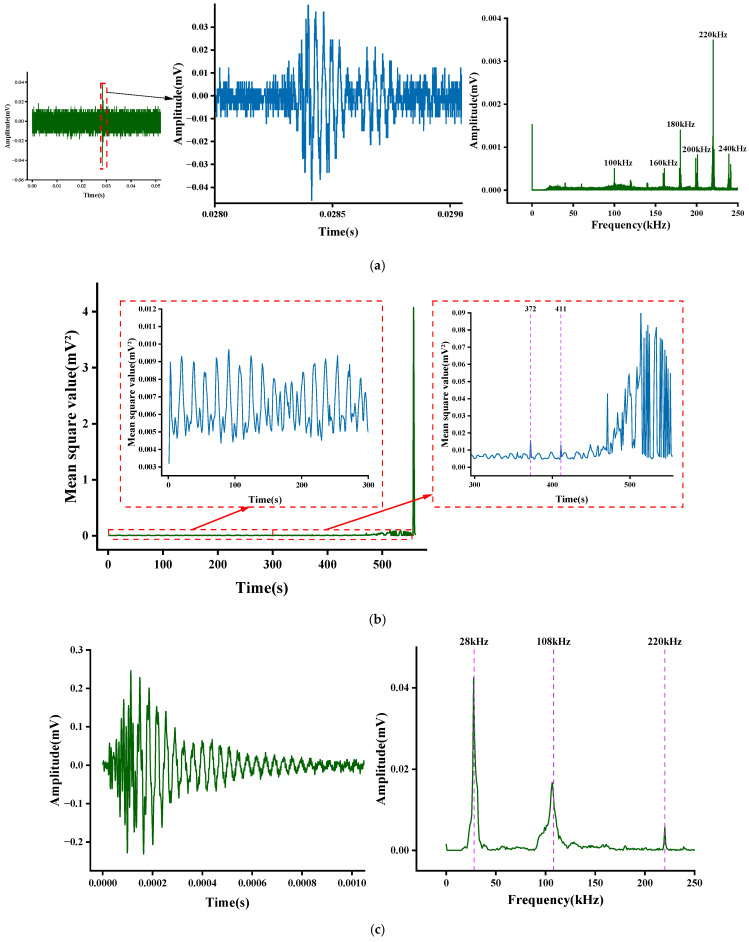
Three abnormal shock waveforms: (**a**) the first type of abnormal waveform; (**b**) the second type of abnormal waveform; and (**c**) the third type of abnormal waveform.

Figure 7 shows the time–frequency domain information for the anomaly waveforms. In layman’s terms, an anomaly waveform represents an impact event in AE, indicating the emergence of abnormal elastic waves within a steady signal. This manifests as an impulse in the time domain; condition monitoring analysis centers on this impact event and its associated parameters. During overcharge, three distinct anomaly waveforms with consistent shapes predominantly emerge. Once initially detected, these waveforms persistently reappear as TR progresses. The first anomaly waveform (Figure 7a) first appears at 57 s, coinciding with the initial abnormal pulse factor during overcharge initiation. Spectral analysis reveals three dominant frequency components at 32 kHz, 28 kHz, and 22 kHz, alongside the ubiquitous 220 kHz frequency present in stable signals. The second waveform (Figure 7b) first appears at 240 s, later than the first type. The initial portion of this waveform exhibits intense yet relatively smooth amplitude fluctuations, followed by smaller oscillations in the latter part. Its primary frequencies are 28 kHz and 32 kHz, indicating the emergence of another type of internal abnormal elastic wave signal distinct from the first category of special waveforms. The third waveform (Figure 7c) primarily occurs when the time-domain characteristic pulse factor and steepness exhibit extremely high abnormal values. First appearing at 439 s, this waveform is relatively regular, with clear frequency components, primarily at 28 kHz and 108 kHz.

**Figure 8:**
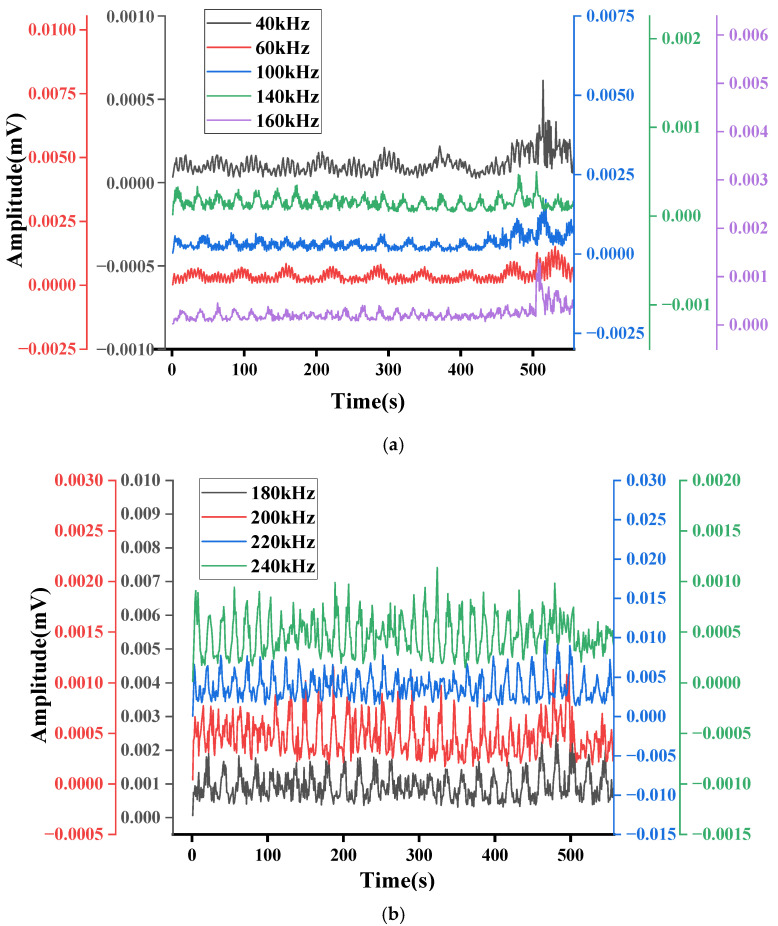
Amplitude variations at different frequencies: (**a**) pulse factor; and (**b**) kurtosis factor.

**Figure 9:**
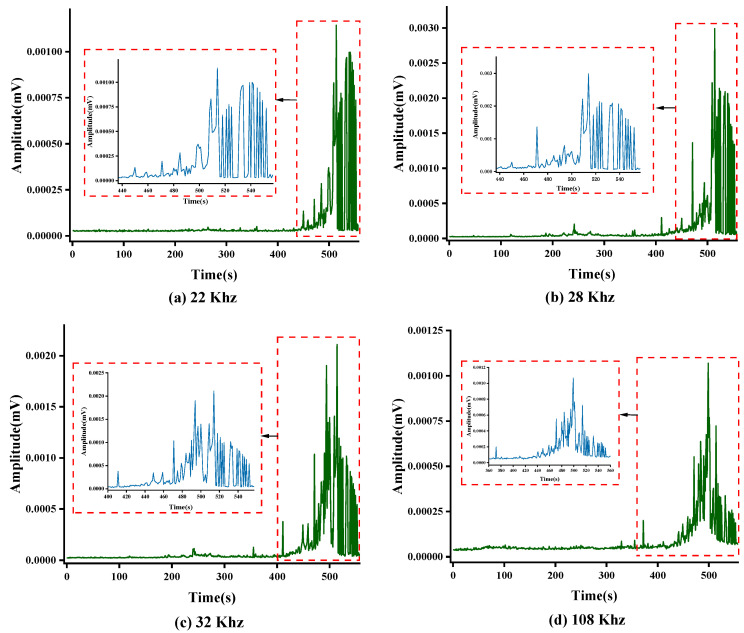
Abnormal waveforms exhibit amplitude variations in frequency.

To identify signal frequencies associated with battery thermal runaway, we analyzed both the consistently high-amplitude frequencies within the acoustic signal spectrum captured during overcharging and the amplitude variations of primary frequencies carried by abnormal waveforms. Each second of the acoustic signal was Fourier transformed into a spectrum. Narrowband frequency domain waveforms were extracted at 1 kHz for the abnormal waveform and at 2 kHz (a 20 kHz harmonic) for the steady signal. The maximum amplitude values were then determined, yielding the curves showing amplitude variation over time at different frequencies, as depicted in Figure 8 and Figure 9. It can be observed that in the time–frequency domain diagram of the stable signal unrelated to the anomaly waveform, the amplitude exhibits periodic variations prior to the battery valve opening. The amplitudes at 40 kHz and 100 kHz frequencies increase starting from 471 s, presenting intense fluctuations at high amplitudes, with sudden spikes in amplitude (Figure 8a). Meanwhile, the amplitudes at 60 kHz and 160 kHz suddenly increase at 505 s before abruptly returning to lower levels. The 140 kHz frequency exhibits periodic low-level variations throughout, except for higher anomalous values at 471 s and 505 s. As shown in Figure 8b, the high-frequency signals at 180 kHz, 200 kHz, 220 kHz, and 240 kHz exhibit consistent periodic variations prior to valve opening.

The four characteristic frequencies identified in this study—22, 28, 32, and 108 kHz—were determined empirically as the dominant frequency components of the three anomalous waveform types observed during overcharge progression (Figure 7). These frequencies are defined here as anomalous dominant frequencies (ADFs): they exhibit non-periodic, monotonically increasing amplitude trends with overcharge progression (Figure 9), in contrast to the quasi-stationary spectral behavior observed at other frequency components, and they emerge exclusively in association with the three anomalous waveform types rather than the steady-state background AE activity. The precise physical mechanisms responsible for generating AE sources at these specific frequencies—whether attributable to gas bubble dynamics, electrode fracture kinematics, or electrolyte decomposition reactions—require dedicated microscopic validation (e.g., in situ X-ray tomography combined with acoustic source localization) and will be systematically investigated in future work. The present study establishes the phenomenological association between these ADFs and TR stage progression as a foundation for that subsequent mechanistic analysis.

As shown in Figure 9, the abnormal waveforms exhibit non-periodic amplitude variations at frequencies of 22 kHz, 28 kHz, 32 kHz, and 108 kHz. These variations resemble the aforementioned time-domain characteristics, confirming that these four frequencies are associated with thermal runaway in lithium-ion batteries. Other frequencies remain unchanged throughout the overcharge process, indicating that they contain fewer thermal-runaway features and exhibit lower sensitivity to thermal-runaway progression.

The three anomalous waveforms described in this study are the most prevalent and typical ones observed during battery overcharging, exhibiting nearly identical waveform characteristics. In reality, various impact signals with distinct waveforms exist, often contaminated by noise-induced interference. Therefore, relying solely on the time–frequency domain features of anomalous waveforms to classify stages of lithium-ion battery TR is unreliable and highly random. Regarding noise handling, the feature extraction process itself within neural networks for acoustic signal data inherently serves as a denoising procedure. As is well known, the collected AE signals represent the superposition of multiple sound sources, including structural vibrations of the battery casing, nucleation and collapse of bubbles in the electrolyte, and fracture of electrode materials. Without additional measurements, such as X-ray tomography or multi-sensor localization, completely separating these sources is challenging. However, the three types of abnormal waveforms identified in this study exhibit sufficiently distinct time–frequency characteristics, with the aim of extracting these abnormal features to provide a basis for subsequent battery abnormality warnings. Importantly, the high accuracy of the classification model (99.87%) indicates that despite the presence of source superposition, the combined AE features carry sufficient discriminative information for reliable TR stage identification. Consequently, this study proposes an intelligent identification method for the early-stage TR of lithium-ion batteries. This approach involves preliminary processing and feature extraction of AE signals, followed by classification of signals from different stages using a trained neural network model.

## 4. TR Phases

### 4.1. Data Process and Model Structure

The raw continuous acoustic signals collected during the experiments are time-varying, one-dimensional, sequential data. While one-dimensional convolution is the most commonly used operation for processing such data, it is highly sensitive to local patterns within the sequence. This limits its effectiveness for feature extraction from large-scale continuous data, as it may lead to the loss of edge-related information. Therefore, the raw acoustic signals were not used directly as input to the classification model. From the above analysis, it is evident that the spectral information of steady and abnormal waveforms in AE signals is relatively clear. The frequency of abnormal waveforms is not affected by noise. Furthermore, as overcharging progresses, although frequencies may shift slightly, the variation patterns of signal amplitudes across frequencies over time can still be effectively extracted. Therefore, this study primarily focuses on processing the spectral information of 0.05 s continuous AE data. The main processing steps are as follows:

1. Divide the time-domain information of the pre-valve-opening signal into 0.05 s time segments. Perform a Fast Fourier Transform (FFT) on each segment to obtain its spectrum, then extract the valid frequency band between 20 kHz and 250 kHz;
2. Divide the spectrum of each time domain segment into frequency bands. Due to the presence of abnormal waveforms, take the maximum amplitude value within the 2 kHz frequency band on either side of the abnormal waveform frequency. If the range is insufficient, use the frequency boundaries of the valid signal. Since the primary frequencies of the abnormal shock waveforms are 22 kHz, 28 kHz, 32 kHz, and 108 kHz, prioritize the frequency bands 20–24 kHz, 26–30 kHz, 30–34 kHz, and 106–110 kHz. The remaining frequency bands are divided equally into n bands, and the maximum amplitude values k0 to kn − 1 are extracted from each band;
3. Take the maximum value $k_{max}$ and minimum value $k_{min}$ of $k_{0}$ to $k_{n-1}$, normalize them, and multiply by 255 to form a grayscale bar, i.e.,:
  (5) $$k_{i}^{\prime }=\frac{k_{i}-k_{min}}{k_{max}-k_{min}}\times 255(i=0,1,2,\ldots ,n-1)$$
4. Connect ${k_{0}}^{\prime }$ − ${k^{\prime }}_{n-1}$ horizontally to form a one-dimensional array (1, n). Continuously extract 0.05 s time-domain segments in chronological order, perform steps 1–3 on each segment, then horizontally connect the resulting arrays into another array (1, n). Vertically concatenate N one-dimensional arrays to form a two-dimensional array (N, n). Each n × n row forms a (n, n) grayscale image. Then, shift down one row to obtain multiple grayscale images of size (N − n, n, n).

Compared with other bands, the band containing the anomalous waveform frequency reflects the abnormal elastic waves emitted by the battery more effectively. Therefore, priority is given to segmenting the frequency components encompassing the anomalous waveform.

Generally, the most valuable information in a spectrum is the frequency with the largest amplitude and its amplitude value, which are used to analyze the dominant frequency. Similarly, taking the maximum amplitude in each frequency band represents the maximum energy in that band’s signal. This serves as a crucial feature for distinguishing abnormal frequencies from others.

After normalization, the signal spectrum from the continuous-time sequence was processed using the same procedure and vertically stacked. To obtain the feature information for each time segment, the image was shifted downward sequentially by one group at a time. This process generates a grayscale image that captures the maximum amplitude variations across different frequency bands over time, serving as the data sample for the neural network model.

**Figure 10:**
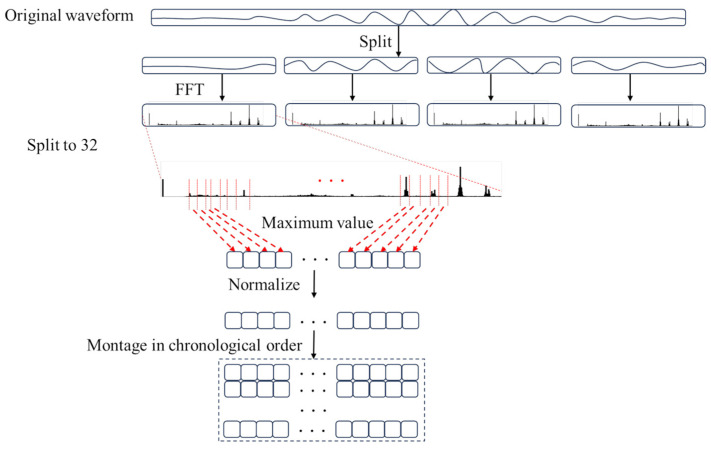
Data processing.

The data processing method proposed in this study simplifies the time–frequency map by essentially decomposing it into time intervals and frequency bands. It extracts the maximum amplitude value from each sub-cell within the decomposed time–frequency map. After processing, the resulting grayscale image contains the maximum-amplitude information across different frequency bands horizontally and the variation in the maximum-amplitude band over continuous time vertically. The data processing flowchart is shown in Figure 10. For the value of n, after multiple configurations were evaluated, including values of 8, 16, 32, 64, and 256 were tested. Convolutional neural networks (CNNs) have proven highly effective for image processing. After training and validation using structurally similar models with corresponding parameters, the accuracy rate initially increased, then declined, peaking at n = 32. Therefore, n = 32 was selected for this study. Through data processing and neural network training, environmental noise outside the main frequency band of the abnormal shock waveform can be effectively suppressed, achieving noise reduction.

The network employed in this study primarily consists of three convolutional layers and two fully connected layers. Detailed network architecture and parameters are presented in Table 2. Each convolutional layer uses a 3 × 3 convolution kernel and adds padding to each data channel. Ensuring identical input and output shapes after convolution. Dropout operations follow the max-pooling layer after the second convolution, the flatten layer, and the fully connected layers. These randomly discard neurons with probabilities of 0.2, 0.2, and 0.3, respectively, for regularization, preventing model overfitting. Since the task of categorizing lithium-ion battery pre-valve stages is a classification problem, the SoftMax activation function is applied to the final output.

### 4.2. Training and Results

This study utilizes PyTorch 2.4.1 + cu118 to construct and configure the structure and parameters of a convolutional neural network. Prior to model training, the final output count is set to 6, dividing the 556-s interval from battery overcharge initiation to valve opening into 6 equal phases. To address boundary attribution issues between adjacent stages, 1-s data segments adjoining each stage were removed. This resulted in 92-s grayscale images per stage after processing. Each stage yielded 1809 grayscale images, from which 1552 were randomly selected for the training set and the remaining 257 for the test set. For training, this study employed an NVIDIA GeForce RTX 4050 Laptop GPU with a batch size of 32. The classification task utilized the commonly applied cross-entropy loss function and Adam optimizer, with a learning rate of 0.0001 over 50 training epochs.

**Figure 11:**
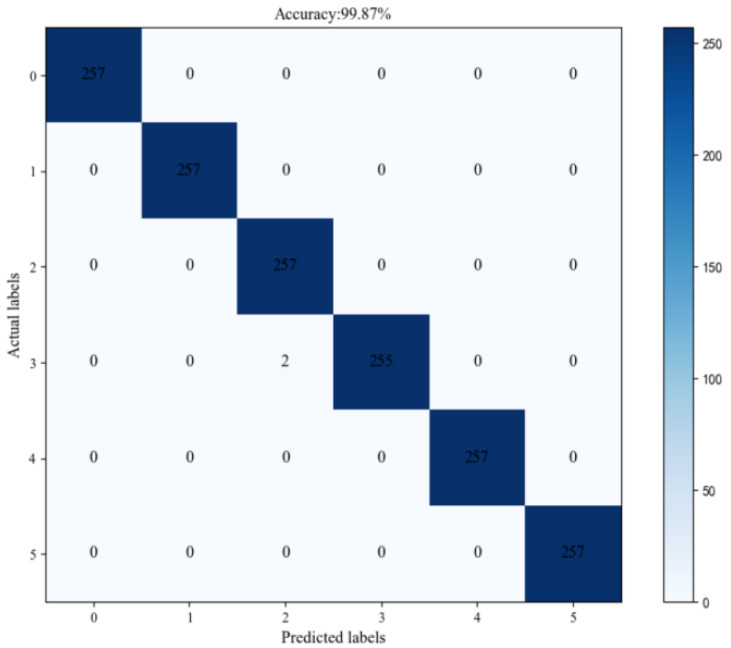
Confusion matrix.

**Figure 12:**
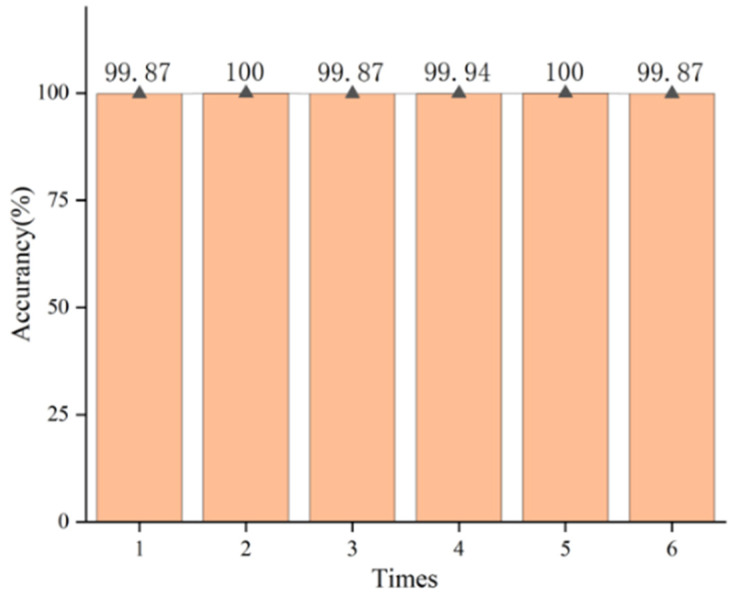
Cross-validation experiment results.

The confusion matrix of the model’s predictions on the test set after training is shown in Figure 11. It shows that the current model achieves a classification accuracy of 99.87% for AE signals, with only two misclassified samples in the grayscale images of the test set, indicating high precision. To validate the effectiveness, six-fold cross-validation was performed, as shown in Figure 12. The model’s predictions across different training and test sets maintained high accuracy, even reaching 100% in some cases. This indicates that the feature extraction process applied to the continuous AE signal data collected from LIBs is effective. Continuous acoustic signals from different stages of battery overcharging contain sufficient feature information. It also demonstrates the strong feature-recognition capability of the current three-layer convolutional neural network for the data-processing methods used in this study and for acoustic signals.

To further validate the superiority of the proposed spectral grayscale CNN approach, we compared it against three conventional machine learning baselines using the same dataset. For each sample, a 96-dimensional hand-crafted feature vector was extracted from four equally divided signal segments, comprising eight time-domain statistics (RMS, variance, peak amplitude, pulse factor, kurtosis, skewness, crest factor, and signal energy) and sixteen frequency-domain features (maximum amplitudes at the four TR-characteristic frequency bands of 22, 28, 32, and 108 kHz; eight steady-state frequency band amplitudes; dominant frequency; spectral centroid; spectral standard deviation; and spectral entropy). Three classifiers were evaluated: support vector machine (SVM) with an RBF kernel, random forest (RF) with 200 estimators, and gradient boosting (GB) with 150 estimators. All classifiers were trained on 80% of the data and tested on the remaining 20%, with stratified sampling to ensure balanced class distribution. Five-fold cross-validation was additionally performed to assess generalization. The comparison results are summarized in Table 3.

As shown in Table 3, all three conventional classifiers achieved test accuracies in the range of 83–87%, with gradient boosting performing best at 86.54%. The proposed spectral grayscale CNN outperforms the best conventional baseline by 13.33 percentage points (99.87% vs. 86.54%), demonstrating the advantage of the two-dimensional frequency-domain representation. Unlike hand-crafted features that rely on predefined frequency bands and statistical descriptors, the CNN-based approach can automatically learn discriminative spectral patterns across both frequency and time dimensions, enabling more precise stage boundaries and higher sensitivity to subtle AE signature transitions during TR incubation.

**Figure 13:**
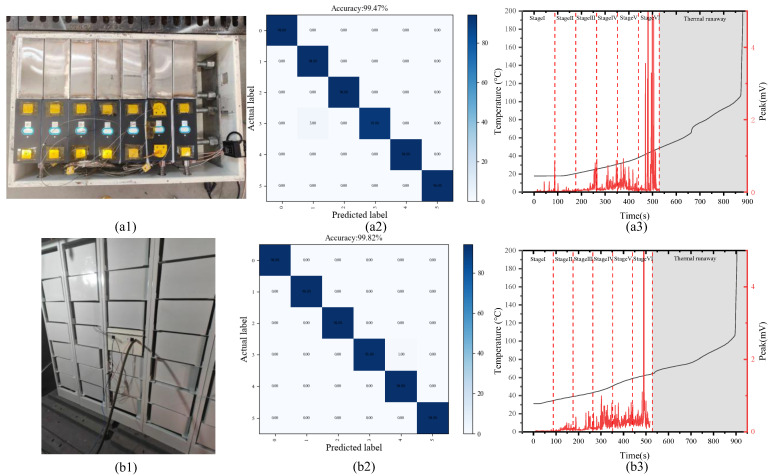
(**a1**–**a3**) simulating the temperature changes and confusion matrix of acoustic emission in the module environment; and (**b1**–**b3**) simulating the temperature changes and confusion matrix of acoustic emissions in energy storage warehouse environment.

Regarding noise processing, convolutional neural networks themselves constitute a denoising mechanism, as they are capable of extracting amplitude changes (including noise) related to time and frequency from sound signals. To verify the generalization ability of the model in different environments, as shown in Figure 13, the research team conducted two repeated experiments in two different scenarios: simulating battery module environments and simulating energy storage warehouses. The average classification accuracy of the test set is 99.47% and 99.82%, respectively, which confirms that the proposed CNN architecture maintains robust recognition performance for continuous acoustic emission data in common practical deployment environments. It should be noted that both the training and testing sets of the main experiment come from the same battery cell, which limits the direct evaluation of cross-cell generalization ability. This limitation has been partially addressed by introducing repeated experiments with different acoustic boundary conditions in different environments; however, subsequent research will use data from independent batteries as a retained test set to rigorously validate cross-battery classification performance. This indicates that the current CNN architecture has achieved a high recognition rate for continuous acoustic emission data before valve opening in common environments, accurately classifying the pre valve-opening stage of lithium-ion batteries and identifying the ultra-early stage of thermal runaway incubation.

In order to verify the advantages of sound signals in monitoring the ultra-early stage of lithium-ion battery TR start-up, Figure 13(a2,b2) show the temperature data collected simultaneously in the experiment and the phase classification achieved by the neural network classification task in this study. It can be observed that the turning points of temperature rise rate occur at 650 s and 530 s in the two environments, respectively. This turning point occurs during the thermal runaway stage after valve opening. Prior to this turning point, the proposed AE-based method achieved high accuracy in determining the current TR stage of the battery, as the AE signal exhibited the first detectable anomaly 100 s after the start of overcharging. This indicates that the temperature inflection points at 650 s and 530 s are advanced by approximately 550 s and 430 s, respectively. In contrast, temperature-based methods can only identify the onset of accelerated TR during the thermal runaway phase, when the internal exothermic reaction has significantly progressed and the window for safety intervention is very limited. These results quantitatively demonstrate the superiority of continuous AE monitoring in the early detection of TR latency, enabling proactive risk management before triggering irreversible chain reactions.

## 5. Conclusions and Limitations

This study experimentally collected continuous AE signals from a large square-case LFP battery undergoing overcharge from 100% SOC until TR. Time–frequency domain signal analysis and feature extraction were performed to establish a mapping relationship between AE waveform characteristics and different evolutionary stages during the early incubation phase of TR. After dimensionality reduction of the one-dimensional AE signals, the normalized spectral signals along the continuous time sequence were stacked vertically. This formed a two-dimensional input dataset containing time–frequency domain feature information, which was fed into a neural network. This approach enabled accurate classification of the incubation stage of TR in LIBs before valve opening. Performance comparisons with traditional external TR detection methods (temperature signals) validated the sensitivity of AE signals to the incubation stage of TR recurrence. Key contributions include:

(1) Continuous AE data were collected at different locations on the surface of LIBs during overcharging until valve opening. The variance, mean square value, pulse factor, and kurtosis of the AE signals were analyzed in the time domain per second. A correspondence was established between changes in the time-domain signal characteristics during the overcharging process and the different stages of TR;

(2) Three distinct abnormal AE waveforms emerged during overcharging. These waveforms first appeared at 57 s, 240 s, and 439 s, respectively, and persisted thereafter, representing the onset of abnormalities, continuous defect generation, and gradual battery failure during overcharging;

(3) Analysis of amplitude variations in primary frequencies during overcharging revealed that the amplitudes of 22 kHz, 28 kHz, 32 kHz, and 108 kHz components in the abnormal waveforms progressively increased with overcharging, effectively reflecting distinct TR stages. In contrast, other frequencies, such as 40 kHz, 60 kHz, 100 kHz, 140 kHz, and 160 kHz, as well as higher frequencies like 180 kHz, 200 kHz, 220 kHz, and 240 kHz, exhibited periodic amplitude variations over time, demonstrating lower sensitivity across different stages of lithium-ion battery TR;

(4) Data processing was applied to the spectrum of continuous AE signals. The maximum spectral features in the temporal sequence were normalized and stitched to form grayscale images, which were used as input sample datasets. A convolutional neural network was constructed to achieve six-stage classification before lithium-ion battery TR initiation, achieving exceptional accuracy and outperforming TR early warning strategies based on physical parameters such as temperature.

Despite the promising results, several limitations should be acknowledged. First, the present study is based on a single battery chemistry (LFP) and a single abuse mode (overcharge); the applicability of the proposed method to other chemistries (e.g., NMC, NCA) and abuse conditions (thermal abuse, mechanical penetration) requires further investigation. Second, the training and test sets were derived from the same cell experiment; cross-cell validation remains to be performed. Regarding methodological transferability, the proposed spectral grayscale CNN framework is inherently chemistry-agnostic in its architecture; however, the specific characteristic frequencies (22, 28, 32, 108 kHz) and their temporal evolution patterns are expected to differ for NMC or NCA chemistries, which exhibit different dominant degradation mechanisms (e.g., oxygen release, phase transitions) generating AE signatures at potentially different frequency ranges. New training data from the target chemistry would, therefore, be required, although transfer learning strategies—pre-training on LFP data and fine-tuning with a small number of target-chemistry samples—could substantially reduce this data acquisition burden. This represents a key direction for extending the proposed method to a broader range of battery types. Third, the current model does not account for the influence of battery aging or capacity fade on AE signal characteristics, which may affect classification performance in fielded systems. Finally, the four anomalous dominant frequencies (22, 28, 32, and 108 kHz) were identified through data-driven spectral analysis rather than derived from first-principles acoustic modeling. While their empirical association with TR stage progression is robust (as evidenced by the 99.87% classification accuracy), the underlying physical generation mechanisms remain to be elucidated. Future work will combine in situ acoustic source localization with post-mortem material characterization to establish the mechanistic basis for these frequency signatures.

For large-scale deployment, several engineering challenges must be addressed:

(1) sensor durability and adhesion reliability under prolonged thermal cycling; (2) signal attenuation and inter-cell acoustic crosstalk in multi-cell battery packs: in a densely packed module, AE waves generated by one cell can propagate to adjacent cells, potentially introducing spurious spectral features that are misattributed to the monitored cell. Mitigation strategies include spatial filtering based on wave arrival time differences across multiple sensors and frequency-domain gating to exclude frequencies dominated by inter-cell propagation modes. These approaches will be evaluated in future pack-level experiments; (3) the computational cost of real-time CNN inference in embedded battery management systems (BMS); and (4) the need for model adaptation across different cell formats and pack configurations.

Future research will focus on: (1) extending the methodology to multi-chemistry datasets and multi-abuse-mode scenarios; (2) developing online real-time AE monitoring systems compatible with commercial BMS; (3) employing transfer learning to reduce training data requirements for new battery types; and (4) validating the approach in multi-cell pack configurations where AE wave propagation introduces additional complexity.

## Figures and Tables

**Table 1:** Comparison of monitoring methods.

Monitoring Mode	Item	Advantages	Disadvantages
External	Temperature	easily	time lag
	Gas	accessible	data latency
	Strain	cost-effective	
Internal	Temperature	direct	sensors with demanding specifications, which are integrated within the battery structure, lead to high overall costs
	Gas	compelling	

**Table 2:** The structure of CNN.

Layer Type	Layer Size	Layer Type	Layer Size
1st Convolutional layer	3 × 3 × 16	Flatten	4096
MaxPooling layer	2 × 2	Dropout layer	0.2
2nd Convolutional layer	3 × 3 × 32	Full-connect layer	128
MaxPooling layer	2 × 2	Dropout layer	0.3
Dropout layer	0.2	Softmax	6
3rd Convolutional layer	3 × 3 × 64		

**Table 3:** Comparison of classification accuracy among different methods for TR stage identification.

Method	CV Accuracy	Test Accuracy
SVM(RBF)	81.82% ± 3.45%	83.65%
Random Forest	82.55% ± 1.78%	83.65%
Gradient Boosting	82.32% ± 2.57%	86.54%

## Data Availability

The original contributions presented in this study are included in the article. Further inquiries can be directed to the corresponding author.
